# The Determination of Normal Vision

**Published:** 1881-02

**Authors:** Francis M. Perkins


### THE DETERMINATION OF NORMAL VISION.

BY FRANCIS M. PERKINS, A. M., M. D.

It often happens that a physician wishes to know whether a patient has normal acuteness of vision; but exactly how to determine its existence or to state in terms of precision what vision exists, he does not always know. The following statement of the method of procedure aims to present it stripped of some points which, though essential to the oculist, are comparatively useless to the general practitioner. The necessary apparatus is a measuring rod and a book of test types. The most convenient measure is a yardstick graduated on one side in English inches, and on the other in centimetres. Such a measure is made by the American Metric Bureau, of Boston, and costs about fifty cents. The best * test types are those of Dr. H. Snellen, of Utrecht; their title is: "Test Types for the Determination of the Acuteness of Vision."

Snellen's Test Types are obtainable from any medical bookseller at a cost of about two dollars. They consist of a series of types, ranging from those visible at two hundred feet to those visible no further than twenty inches. In the latest editions, over each size of type are figures indicating in metres, or fractions thereof, the greatest distance at which that sized type is visible to an eye having normal vision. Remembering that one metre equals 3.28 English feet, or 39.37 inches, the conversion of these figures into their English equivalents is easy.

The pages of Snellen containing *letters* marked D=60 and D=3, and those with letters of intermediate size, should be cut out and pasted on a large sheet of card-board. This should be hung on the wall of your office which has the best illumination,

* Test types intended to be used as are Snellen's are printed by a number of opticians, but in accuracy of form of the letters and clearness of impression they are not equal to the imported Snellen's types. Moreover, Snellen's book contains reading matter in all the principal languages. For these reasons Snellen's book is to be preferred and reference is confined to it.

the bottom of the card being about four feet from the floor. From the card, measure a distance equal to six metres (twenty feet). Should the size of your office not give you this distance, take the nearest approach to it in *whole metres* that you can obtain. Next note with a pencil mark, or otherwise, on the wall, this distance, so that you will always know it without the necessity of measuring more than once. Never guess at this distance just before making an examination. Place the patient so that when he faces the card his eyes shall be exactly opposite to the mark on the wall. Hold in front of one eye a piece of dark colored card-board or blotting paper, in order that this eye may be excluded from participation in vision while you are examining the other. Be careful that no pressure is made upon the eye, and that the edge of the card is held closely in contact with the nose. The reason for the first precaution is, that if pressure is made, never so slightly, on the eye, the patient will tell you, when you commence to take the vision of this eye, that he has a "haze over the eye." The reason for the second precaution is, that many people will try, by turning the head, to look out of that eye which you are excluding. Unless you hold the card close against the nose they will succeed in doing this, and you may record the vision of the eye you suppose to be excluded. instead of that of the other one.

These precautions are especially to be observed in the case of children, and failure to observe them may vitiate the accuracy of your conclusions. In this portion of the examination the patient should not be allowed to hold the card: nor in an examination laying any claim to accuracy should the hand of either examiner or patient be used to cover the eye. If you have any reason to suppose that vision is less acute in one eye than in the other, commence with the worst eye.

Having covered one eye, ask your patient to name aloud the letters, commencing with the largest. You must not begin by saying: "Do you see the letter on the top of the card?" mentioning at the same time its name. By adopting this plan, a patient with a good memory has been known to repeat after the examiner all the letters on the card; when, in point of fact, he never saw one of them. Merely request that the letters be named aloud in order of size, until a mistake is made or the patient says he can see no more. Never correct any mistakes. By requesting a repetition of a line in which mistakes are made, you can easily satisfy yourself whether these are due to carelessness or to inability to see the letters. Some patients, in compliance with your request for the letters, will say, complacently, "I can see all the letters on that card, Doctor!" You may, in such a case, introduce a variation, by asking for the bottom line. This they may be unable to read, and after an unsuccessful attempt, will comply with your original request and commence with the top letter.

Suppose a patient at a distance of six metres from the card reads with his right eye the letters bearing above them "D=6." He will then have the power of seeing at six metres letters that an eye with normal vision sees at that distance. You record his vision in the form of a fraction, viz.: "R E $V=\frac{6}{6}$," where the denominator shows the size of the type read and the numerator the distance between the eye and the letters. If the distance is five metres, and the type seen is "D=5," then "$V=\frac{5}{5}$."

Of course, these fractions might be reduced to their lowest terms, and you might correctly say, "V=1." But expressing the vision by an unreduced fraction shows at a glance the distance used in making the examination; consequently the fraction should not be reduced.

Should you find an eye able, at five metres, to read "D=4.' you will have a case of more than ordinary sharpness of vision, and you will record "$V=\frac{5}{4}$." These cases of unusual acuity of vision are rare, and when you think you have one, always take the precaution of having the letters repeated backward.

Passing now to the other eye; suppose that at six metres nothing smaller than "D=24" is read. Acuity of vision is diminished in this eye, and you say "$V=\frac{6}{24}$." Reduced to its lowest terms, this fraction means that vision is only one quarter of normal. But for the reasons above given, you allow the fraction to remain unreduced.

If you wish to be very exact, you may modify the fraction expressing vision, as follows: — Suppose "D=18" is read at six metres, but imperfectly, *i. e.*, only about one-half of the total number of letters is read, the others being misnamed. You may then say, "$V=\frac{6}{18}$? (fifty per cent.") Meaning, that though vision is *better than* $\frac{6}{24}$ it is not exactly $\frac{6}{18}$. In like manner, if one quarter or two-thirds be read correctly, you will say $V=\frac{6}{18}$? (twenty-five per cent.,) or $\frac{6}{18}$? (sixty-six per cent.) as the case may be. This may be deemed a refinement of precision; yet it takes but little trouble, and the record so made may be very useful at some subsequent examination, by showing increased or diminished sharpness of vision greater or less than that shown by the gain or loss of an entire line.

When the acuity of vision is diminished to such a degree that at your standard distance of six, five, or four metres (depending on the size of your office) even the top letter (D=60) cannot be distinguished, you cause the patient to walk slowly up to the card, making him stop every foot or two until he is near enough to tell the top letter.

If he has to come within one metre of the card to do this, then $V=\frac{1}{60}$; if within half a metre, then $V=\frac{0.50}{60}$. You will sometimes find vision perfect with one eye, while with the other it may be down to $\frac{2}{60}$, or lower.— *The American Specialist.*

